# Foxp3 expression in human cancer cells

**DOI:** 10.1186/1479-5876-6-19

**Published:** 2008-04-22

**Authors:** Vaios Karanikas, Matthaios Speletas, Maria Zamanakou, Fani Kalala, Gedeon Loules, Theodora Kerenidi, Angeliki K Barda, Konstantinos I Gourgoulianis, Anastasios E Germenis

**Affiliations:** 1 Cancer Immunology Unit, Department of Immunology and Histocompatibility, School of Medicine, University of Thessaly, University Hospital of Larissa, GR-411 10 Larissa, Greece; 2 Department of Respiratory Medicine, School of Medicine, University of Thessaly, University Hospital of Larissa, GR-411 10 Larissa, Greece

## Abstract

**Objective:**

Transcription factor forkhead box protein 3 (Foxp3) specifically characterizes the thymically derived naturally occurring regulatory T cells (Tregs). Limited evidence indicates that it is also expressed, albeit to a lesser extent, in tissues other than thymus and spleen, while, very recently, it was shown that Foxp3 is expressed by pancreatic carcinoma. This study was scheduled to investigate whether expression of Foxp3 transcripts and mature protein occurs constitutively in various tumor types.

**Materials and methods:**

Twenty five tumor cell lines of different tissue origins (lung cancer, colon cancer, breast cancer, melanoma, erythroid leukemia, acute T-cell leukemia) were studied. Detection of *Foxp3 *mRNA was performed using both conventional RT-PCR and quantitative real-time PCR while protein expression was assessed by immunocytochemistry and flow cytometry, using different antibody clones.

**Results:**

*Foxp3 *mRNA as well as Foxp3 protein was detected in all tumor cell lines, albeit in variable levels, not related to the tissue of origin. This expression correlated with the expression levels of IL-10 and TGFb1.

**Conclusion:**

We offer evidence that *Foxp3 *expression, characterizes tumor cells of various tissue origins. The biological significance of these findings warrants further investigation in the context of tumor immune escape, and especially under the light of current anti-cancer efforts interfering with Foxp3 expression.

## Background

The transcription factor forkhead box protein 3 (Foxp3) is considered to be a master control gene of the function of thymically derived naturally occurring regulatory T cells (Tregs) [1]. Due to the Tregs lineage specification by Foxp3, its tissue expression primarily by lymphoid tissues (thymus, spleen and lymph nodes) is expected and it has been well documented [1,2]. Despite, however, the scarcity of information, Foxp3 expression by other normal tissues has also been observed, albeit to a far lesser extent [3]. Moreover, induction of Foxp3 expression can occur intrinsically in peripheral Foxp3- T cells [4], while peripheral activated CD4+CD25- and CD8+CD25- T cells can acquire a regulatory function by expressing Foxp3 [5,6]. Since the factors inducing Foxp3 expression in the above T cell populations remain unknown, we hypothesized that a similar induction could take place in other types of cells such as tumor cells. In support of the above, a very recent publication describes the expression of Foxp3 in pancreatic carcinoma cells providing evidence that this could be an important tumor escape mechanism [7]. To this end, this study was scheduled to investigate whether expression of Foxp3 transcripts and mature protein is confined to pancreatic carcinoma or can occur constitutively in other tumor types as well as whether it might be repressed as a result of promoter hypermethylation, as is the case with several other genes including many associated with a tumor suppressor function [8]. We provide unequivocal evidence that Foxp3 is expressed both at the transcript and protein level by tumor cells of various types.

## Materials and methods

### Tumor cells

Twenty two tumor cell lines of various tissue origins, kindly donated by collaborators (Table 1), as well as 3 cancer cell lines recently established, as previously described [9], from tumor cell suspensions collected from lung adenocarcinoma patients submitted to surgery (PGEGE, PKAKI and PINTZ), were studied. All cell lines were kept frozen and upon thawing they were maintained in complete medium (CM) that consisted of Iscove's medium (Gibco Laboratories, Grand Island, NY, USA), 10% FCS (Gibco), 100 μM Minimal Essential Aminoacids (Gibco), 100 U/mL penicillin, 100 μg/mL streptomycin (Gibco), 2 mM glutamine (Gibco) and 50 μM 2-mercaptethanol (Gibco), supplemented with hydrocortisone, 17β-estradiol, sodium selenite, insulin and transferrin (HITES solution) as described by Carney et al [10], at 37°C in 8% CO_2_.

**Table 1:** Cancer cell lines used in the study.

Cancer cell lines	Type of cancer
CALU-1, CALU-6, GILI, ONET, SK-LU-1, NCI-H441, NCI-H460, NCI-H596, NCI-H661, NCI-H520, PGEGE, PKAKI, PINTZ	Lung cancer
HCA 2.6, HCA 3.2	Colon cancer
MCF7, T47D, HBL-100p40, BT20, MDAMB231	Breast cancer
GERL, DAJU 2.7, MEL272,	Melanoma
K562	Erythroid leukemia
JURKAT	Acute T cell leukemia

To explore whether the expression of Foxp3 by cancer cells is affected by culture with the HITES solution, experiments were undertaken using parallel cultures of tumor cell lines, in the presence and in the absence of HITES solution, i.e. at corticosteroid concentrations used to inhibit development of lymphocytes, for a period of 3 weeks, allowing at least 10–12 cell divisions. All experiments were repeated three times.

### Foxp3 mRNA expression

*Foxp3 *mRNA expression was examined in all cases using both conventional PCR (RT-PCR) and quantitative real-time PCR (qRT-PCR), after total RNA isolation from the tumor cell lines and reverse transcription to cDNA, as previously described [11], qRT-PCR was performed using the automated thermocycler RotorGene 6000 (Corbett Life Science, Sydney, Australia), the SYBR Supermix kit (Invitrogen, Paisley UK) and the RT^2 ^PCR Primer Set for *Foxp3 *(SuperArray, USA). β_2_-Microglobulin (β_2_-M) was used as a reference gene (RT^2 ^PCR Primer Set, SuperArray) [12,13]. The qRT-PCR thermocycling conditions for *Foxp3 *were: 10 min at 95°C initial hold, followed by 40 cycles of denaturation at 95°C, annealing at 60°C and extension at 72°C all for 15 sec. The qRT-PCR thermocycling conditions for β_2_-M were: 10 min at 95°C initial hold, followed by 40 cycles of denaturation at 95°C for 15 sec, annealing/extension at 60°C for 60 sec. Relative expression was analyzed using the Rotor Gene software (Ver. 6) and is presented as a multiple of the gene expression in one fibroblast line isolated during the development of the cancer cell lines. EBV-transformed B cells were used as negative controls, whereas a CD4+ Treg clone (kindly provided by Sophie Lucas, Brussels, Belgium) and PHA blasts were used as positive controls. For RT-PCR amplifications, 20 μM of the following primer sets were used for β-actin, forward 5' GGCATCGTGATGGACTCCG 3' and reverse 5' GCTGGAAGGTGGACAGCGA 3' and the RT^2 ^PCR Primer Set for *Foxp3 *(SuperArray), in a total reaction volume of 25 μL. Thermocycling conditions (PTC-200, MJ Research, Watertown-Mass., USA) included for β-actin 21 cycles of denaturation at 94°C, annealing at 68°C and extension at 72°C, all for 1 min, and for *Foxp3*, 31 cycles of denaturation at 95°C, annealing at 60°C and extension at 72°C, all for 15 sec.

### IL-10 and TGFb1 mRNA expression

*IL-10 and TGFb1 *mRNA expression was examined using both conventional PCR (RT-PCR) for IL-10 and quantitative real-time PCR (qRT-PCR) for TGFb1 using cDNA prepared as above. For TGFb1, the RT^2 ^PCR Primer Set from SuperArray was used, and the qRT-PCR thermocycling conditions were: 10 min at 95°C initial hold, followed by 40 cycles of denaturation at 95°C for 15 sec and annealing/extension at 60°C for 60 sec. *β*_2_*-M *was used as a reference gene as for *Foxp3 *and the relative expression was analyzed and presented as above. For RT-PCR amplifications of IL-10, 20 μM of the RT^2 ^PCR Primer Set for *IL-10 *(SuperArray) was used in a total reaction volume of 25 μL. Thermocycling conditions included 30 cycles of denaturation at 95°C for 15 sec, annealing at 60°C for 15 sec and extension at 72°C for 30 sec. IL-10 expression is presented as a semiquantitative measurement obtained by calculating the intensity quotient for the gene and *β-ac*tin [14] and then normalizing to that of a fibroblast cell line.

### Foxp3 protein expression

To determine whether the expression of *Foxp3 *mRNA results in the production of a mature protein, tumor cell lines were examined by immunohistochemistry and by flow cytometry for intracellular expression of Foxp3, using different antibody clones. Immunostaining was performed according to a previously published protocol with slight modifications [15]. Sections were fixed in 4% paraformaldehyde, blocked in 5% normal mouse serum (eBioscience), and incubated with a mouse monoclonal biotinylated anti-Foxp3 antibody against the amino terminus of the human Foxp3 protein (dilution 1/250) (clone 236A/E7, eBioscience, San Diego, USA), followed by goat anti-mouse secondary antibody and dextran coupled with peroxidase molecules (EnVision Detection System, Peroxidase/DAB, Rabbit/Mouse, Dako). DAB (EnVision Detection System, Dako) was used as peroxidase substrate, while endogenous peroxidase activity was blocked directly after the fixation step with 1% of H_2_O_2 _in 1xTBS. Sections were counterstained with Mayer hematoxylin (Merck).

Flow cytometric detection of intracellular Foxp3 expression was performed using an anti-Foxp3 monoclonal antibody (clone PCH101, eBioscience) labeled with phycoerythrin and the corresponding IgG2a isotypic antibody according to the manufacturer. In each analysis, approximately 10^6 ^tumor cells were used, and approximately 50–100 × 10^3 ^events were acquired and analyzed on the basis of Foxp3 positivity using the CXP program (Beckman Coulter, USA). Positivity was determined on the basis of the Mean Fluorescence Index (MFI) for staining with Foxp3, against the staining observed with the corresponding isotypic antibody.

### Statistical analysis

Expression values are presented as raw values or as mean ± SD. Spearman's bivariate correlation was used to identify correlations (Pearson's correlation and significance is presented) with the statistical software SPSS for Windows (version 11.5).

## Results and discussion

**Figure 1:**
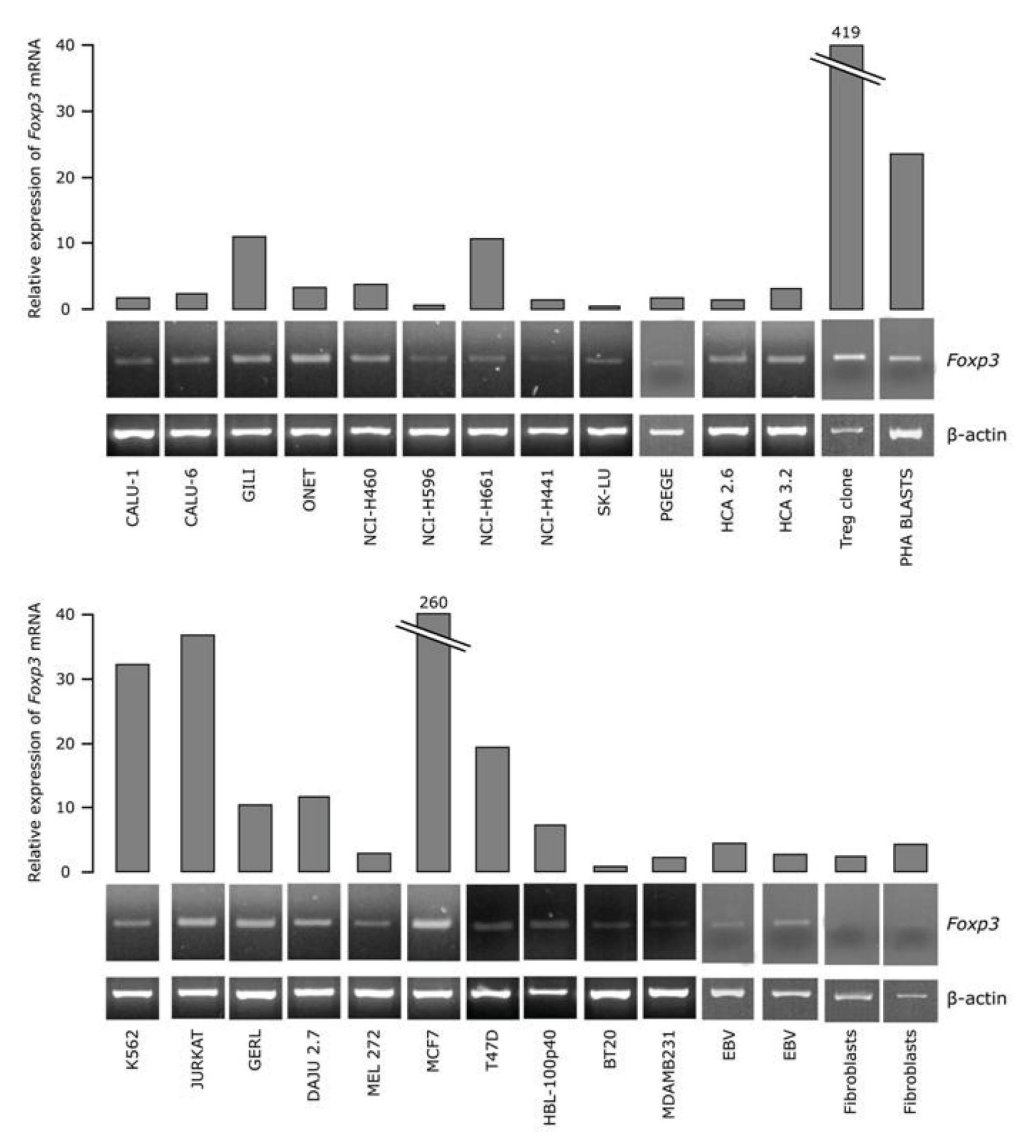
Expression of *Foxp3 *mRNA in tumor cell lines by qRT-PCR (histograms on the top) and RT-PCR (electrophoretic plots in the bottom).

Expression of *Foxp3 *mRNA was revealed in all tumor cell lines studied (Fig. 1). Relative expression values for tumor cells varied widely between 0.5 and 260 (17.4 ± 53.4) and were significantly higher than those of fibroblasts (1.9 ± 1.6) and EBV-transformed B cells (3.5 ± 1.2). This expression did not relate to the tissue of origin of each line. The highest expression levels of Foxp3 observed by tumor cells was with the breast cancer line MCF7 (260), which expressed at least half as much Foxp3 as the Treg clone (419) did, and at least ten times more than a population of PHA blasts (23.5). This expression level indicates that Foxp3 transcripts are present in a sufficiently high number in tumor cells and caution should be exerted when detection of Foxp3 mRNA expression in surgical tumor samples is used as an index of tumor infiltration by Tregs.

**Figure 2:**
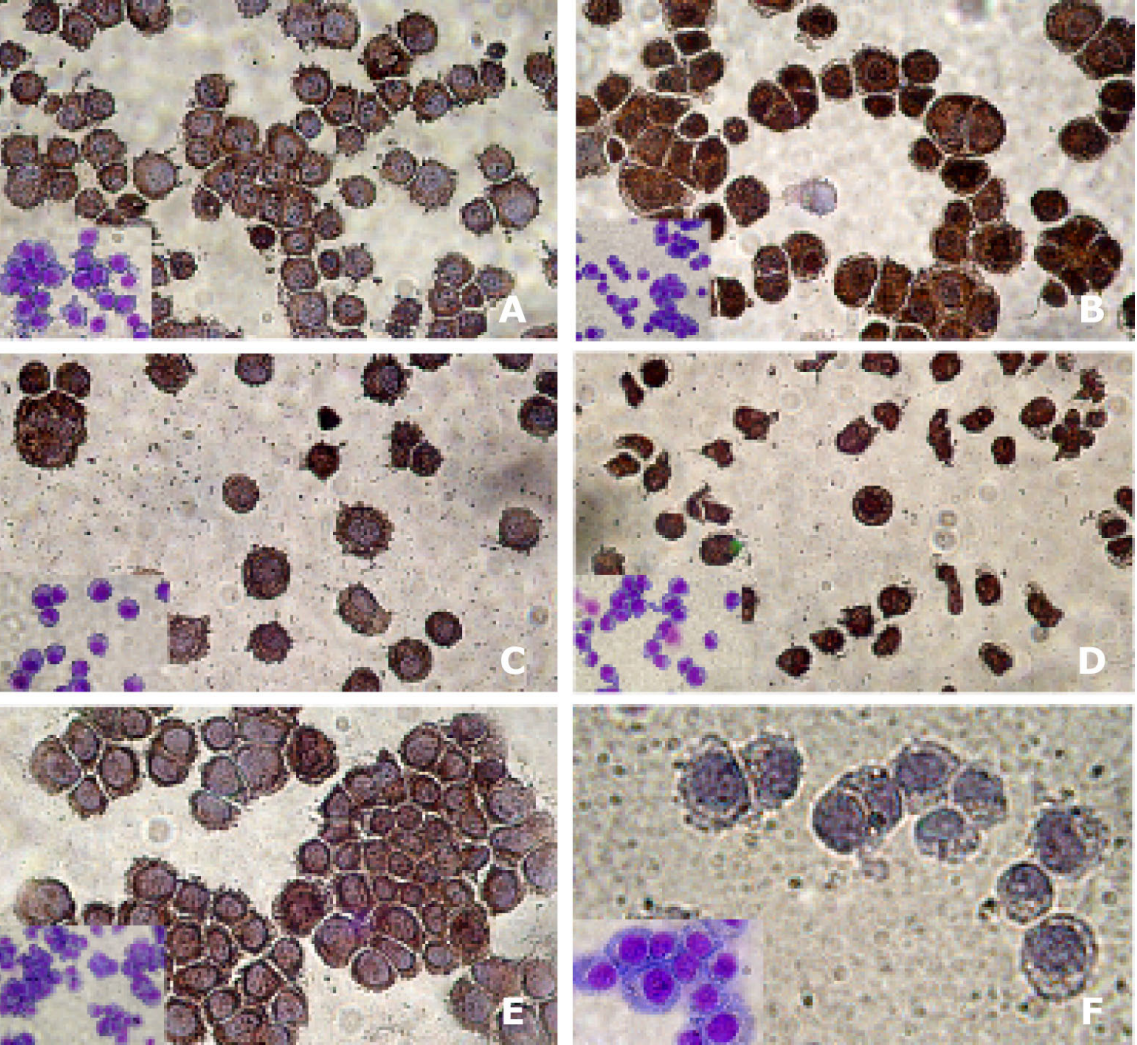
**Immunohistochemical staining of tumor cell line cytospins.** A: Melanoma (GERL); predominant cytoplasmic expression. B: Lung adenocarcinoma (GILI); cytoplasmic and nuclear expression. C: Colon adenocarcinoma (HCA 2.6); predominant cytoplasmic expression. D: T Lymphoblastic leukemia (JURKAT); cytoplasmic and nuclear expression. E: Breast adenocarcinoma (MCF7); predominant cytoplasmic expression. F: Not expressing EBV-transformed B cells. Inserts represent May-Gruvald-Giemsa staining of the corresponding cell lines.

All tumor lines tested, exhibited distinct staining profiles (Fig. 2). Heterogenous subcellular localization of Foxp3 (predominantly perinuclear cytoplasmic and/or mainly nuclear) was observed in lines of epithelial as well as of non-epithelial origin, while EBV-transformed B cells were devoid of staining. This finding warrants further attention with respect to the possible functional consequences of nuclear and/or perinuclear cytoplasmic Foxp3 expression by cancer cells. In particular, under the light of recent findings revealing the physical interaction of Foxp3 with the nuclear factor of activated T cells (NFAT), it appears that at least with respect to its nuclear localization, Foxp3 plays a pivotal role in the formation of nuclear complexes that are important to regulate the transcription of several target genes [16], that confer to Tregs their suppressive function [17].

**Figure 3:**
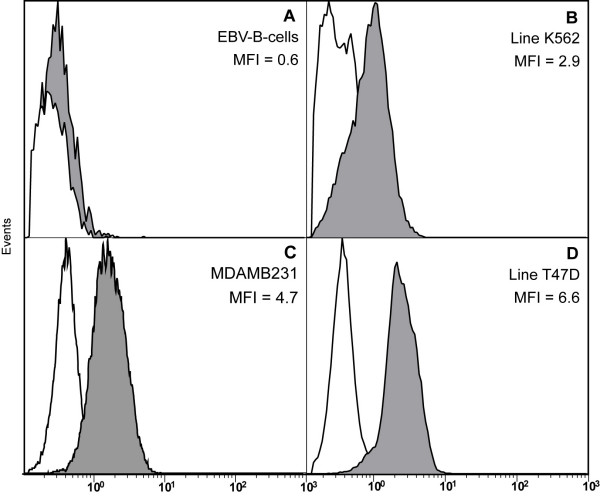
**Flow cytometric detection of Foxp3 expression in various cell lines.** (A) negative control (EBV-transformed B cells), (B) an erythroid leukemia cell line with low expression, and (C+D) breast cancer cell lines with moderate and high expression. The white underlaid plot represents staining with the isotype.

Flow cytometric analysis confirmed the results of immunohistochemistry regarding Foxp3 protein expression. Intracellular Foxp3 was examined in 15 tumor lines, all of which expressed the protein, whilst none of the EBV-transformed B cells and fibroblast lines used as controls displayed any Foxp3 staining (Fig. 3). The mean fluorescence intensity (MFI) varied between 2.07 and 13.90 allowing the discrimination of the tumor cells into three patterns of distinct staining intensity. The first (n = 3), with an MFI 2.07–2.92, the second (n = 9) with an MFI 3.79–4.87 and the third (n = 3) with an MFI >5. The protein expression levels related weakly to the Foxp3 mRNA expression levels.

Corticosteroids significantly increase *Foxp3*-as well as *IL10*- mRNA expression in un-stimulated peripheral blood CD4+ T cells [18]. No difference in *Foxp3 *mRNA expression levels was detected when cancer cell lines (n = 3) were cultured with or without HITES, for a period of 3 weeks (10–12 cell divisions). This difference between cancer cells and CD4+ T cells might represent a mechanism utilized by the first to escape from corticosteroid-regulated death mediated by GITR as it happens in T cells [19].

Following the recent finding that Foxp3 is expressed by pancreatic carcinoma cells [7], our study clearly demonstrates that its expression is not restricted to this particular type of tumor but seems to characterize many other tumors not only of epithelial (e.g. lung, breast, colon) but also of other tissue origins (melanoma, leukemia). Whether Foxp3 expression by tumor cells is directly related to carcinogenesis or results indirectly by activation of its normally silent gene, is questionable. Fibroblasts used as controls in our study exhibited insignificant levels of *Foxp3 *transcripts that is in accordance to the results of Hinz et al [7] showing no Foxp3 expression by normal pancreatic duct epithelial cells. However, Christodoulou et al [20] have shown that normal salivary gland epithelial cells express Foxp3 mRNA, as did three neoplastic lines of epithelial origin, but not human umbilical vein endothelial cells. Foxp3 induction pathways (e.g. those of IFN-γ, TLRs and others) that, along with the TcR-mediated one, are functioning in Tregs [21], could be implicated in cancer and/or transforming cells. In fact, the recently shown induction by TGF-β [7] seems to be a key facet of the complex role of TGF-β in cancer biology [22].

The varying levels of Foxp3 mRNA expression detected in tumor cells raises a serious issue concerning the use of the detection of *Foxp3 *mRNA expression in surgical tumor samples as an index of tumor infiltration by Tregs [23-25]. Thereby, conclusions derived from such studies about the relationship between Tregs and cancer might be misleading [26,27]. This variation however, might also imply a different role for Foxp3 expression by tumor cells. A recent finding in breast cancer cells proposes Foxp3 as an important suppressor for human breast cancer [28]. Functional somatic mutations, and down-regulation of the FOXP3 gene, were commonly found in human breast cancer samples and although this also correlated with HER-2/ErbB2 overexpression it was clearly lower than that of normal breast tissue [28]. Whether the presence of such mutations could account for the very low expression levels observed with some tumor lines in our study remains to be investigated as does confirmation that Foxp3 is a tumor suppressor molecule.

**Figure 4:**
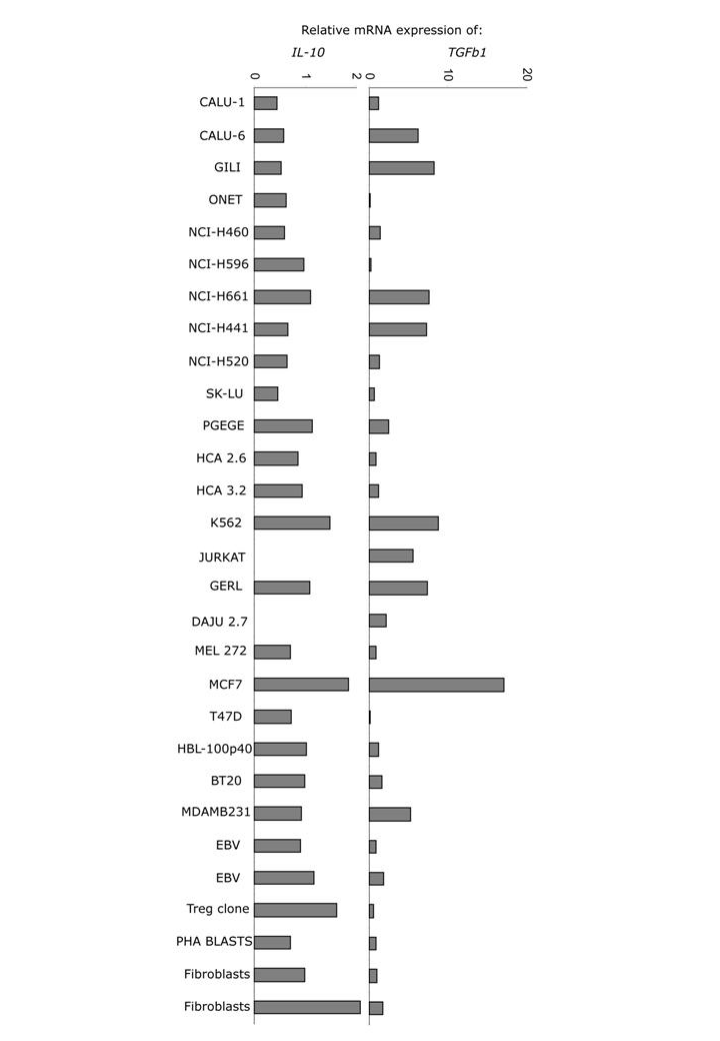
Expression of *IL-10 *(left) and *TGFb1 *(right) mRNA in tumor cell lines.

In assessing further whether Foxp3 mRNA expression detected in tumor cells had any putative functional significance for the tumor cells, we assessed the levels of mRNA expression for IL-10 and TGFb1 (Fig 4). Both IL-10 (0.7 ± 4.2) and TGFb1 (3.6 ± 4.2) were expressed by nearly all tumor cells. Interestingly, these expression levels also correlated with Foxp3 mRNA expression (Foxp3 vs TGFb1: Pearson's correlation = 0.74, significance= 0.0001; Foxp3 vs IL-10: Pearson's correlation = 0.529, significance = 0.009; TGFb1 vs IL10: Pearson's correlation = 0.499, significance = 0.015). Whether, these relations play any significant role in conferring to the tumors the ability to evade immunity remains to be elucidated.

Based on the finding that Foxp3 expression by pancreatic carcinoma results in inhibition of proliferation and possibly not of activation of naïve CD4+ CD25- T cells, Hinz et al [7] propose that this might represent a tumor escape mechanism. Our results, however, uncovering Foxp3 expression as a generalized feature of tumor cells, indicate that the determination of its functional consequences requires further elucidation, especially in the context of current anti-cancer efforts to control the pathogenetic action of Tregs, mainly those interfering with Foxp3 expression [29].

## Conclusion

This study provides clear evidence that cancer cells of various types express a transcript for *Foxp3 *as well as the mature protein. This finding can be of utmost significance under the light of Tregs being implicated in carcinogenesis and ongoing efforts towards the development of anticancer approaches specifically inhibiting the expression and/or function of Foxp3 by tumor-associated Tregs

## Competing interests

The authors declare that they have no competing interests.

## Authors' contributions

VK and AEG equally contributed in the initial idea, design and coordination of the study, and wrote the manuscript. MS coordinated the qRT-PCR experiments, performed the immunohistochemistry, and participated in discussion of the results. MZ and FK performed culture and flow cytometry experiments. MZ, GL and AB performed qRT-PCR experiments. TK collected the clinical samples and KIG overlooked the clinical aspects of the study. All authors read and approved the final manuscript.
